# Amorphous SiO2 nanoparticles promote cardiac dysfunction via the opening of the mitochondrial permeability transition pore in rat heart and human cardiomyocytes

**DOI:** 10.1186/s12989-020-00346-2

**Published:** 2020-05-07

**Authors:** Omar Lozano, Christian Silva-Platas, Héctor Chapoy-Villanueva, Baruc E. Pérez, Jarmon G. Lees, Chrishan J. A. Ramachandra, Flavio F. Contreras-Torres, Anay Lázaro-Alfaro, Estefanía Luna-Figueroa, Judith Bernal-Ramírez, Aldemar Gordillo-Galeano, Alfredo Benitez, Yuriana Oropeza-Almazán, Elena C. Castillo, Poh Ling Koh, Derek J. Hausenloy, Shiang Y. Lim, Gerardo García-Rivas

**Affiliations:** 1 Tecnologico de Monterrey. Escuela Nacional de Medicina y Ciencias de la Salud, Cátedra de Cardiología y Medicina Vascular, Monterrey, Mexico; 2 Tecnologico de Monterrey. Centro de Investigación Biomédica, Hospital Zambrano-Helión, San Pedro Garza-García, Mexico; 3 Departments of Medicine and Surgery, University of Melbourne, Melbourne, Victoria Australia; 4 O’Brien Institute Department, St Vincent’s Institute of Medical Research, Melbourne, Victoria Australia; 5 National Heart Research Institute Singapore, National Heart Centre Singapore, Singapore, Singapore; 6 Cardiovascular and Metabolic Disorders Programme, Duke-NUS Medical School, Singapore, Singapore; 7 Tecnologico de Monterrey, Escuela de Ingeniería y Ciencias, Monterrey, Mexico; 8 Department of Physics and Astronomy, The University of Texas at San Antonio, San Antonio, USA; 9 Yong Loo Lin School of Medicine, National University Singapore, Singapore, Singapore; 10 The Hatter Cardiovascular Institute, University College London, London, UK; 11 Cardiovascular Research Center, College of Medical and Health Sciences, Asia University, Taichung City, Taiwan

**Keywords:** Mitochondria, Silica nanoparticles, Oxidative stress, Cardiotoxicity, Permeability transition, Heart, Calcium overload

## Abstract

**Background:**

Silica nanoparticles (nanoSiO_2_) are promising systems that can deliver biologically active compounds to tissues such as the heart in a controllable manner. However, cardiac toxicity induced by nanoSiO_2_ has been recently related to abnormal calcium handling and energetic failure in cardiomyocytes. Moreover, the precise mechanisms underlying this energetic debacle remain unclear. In order to elucidate these mechanisms, this article explores the ex vivo heart function and mitochondria after exposure to nanoSiO_2_.

**Results:**

The cumulative administration of nanoSiO_2_ reduced the mechanical performance index of the rat heart with a half-maximal *inhibitory* concentration (IC_50_) of 93 μg/mL, affecting the relaxation rate. In isolated mitochondria nanoSiO_2_ was found to be internalized, inhibiting oxidative phosphorylation and significantly reducing the mitochondrial membrane potential (ΔΨ_m_). The mitochondrial permeability transition pore (mPTP) was also induced with an increasing dose of nanoSiO_2_ and partially recovered with, a potent blocker of the mPTP, Cyclosporine A (CsA). The activity of aconitase and thiol oxidation, in the adenine nucleotide translocase, were found to be reduced due to nanoSiO_2_ exposure, suggesting that nanoSiO_2_ induces the mPTP via thiol modification and ROS generation. In cardiac cells exposed to nanoSiO_2_, enhanced viability and reduction of H_2_O_2_ were observed after application of a specific mitochondrial antioxidant, MitoTEMPO. Concomitantly, CsA treatment in adult rat cardiac cells reduced the nanoSiO_2_-triggered cell death and recovered ATP production (from 32.4 to 65.4%). Additionally, we performed evaluation of the mitochondrial effect of nanoSiO_2_ in human cardiomyocytes. We observed a 40% inhibition of maximal oxygen consumption rate in mitochondria at 500 μg/mL. Under this condition we identified a remarkable diminution in the spare respiratory capacity. This data indicates that a reduction in the amount of extra ATP that can be produced by mitochondria during a sudden increase in energy demand. In human cardiomyocytes, increased LDH release and necrosis were found at increased doses of nanoSiO_2_, reaching 85 and 48%, respectively. Such deleterious effects were partially prevented by the application of CsA. Therefore, exposure to nanoSiO_2_ affects cardiac function via mitochondrial dysfunction through the opening of the mPTP.

**Conclusion:**

The aforementioned effects can be partially avoided reducing ROS or retarding the opening of the mPTP. These novel strategies which resulted in cardioprotection could be considered as potential therapies to decrease the side effects of nanoSiO_2_ exposure.

## Introduction

Silica nanoparticles (nanoSiO_2_) have been widely studied for biomedical and biotechnological applications [44], specifically those of amorphous nature, which arise from several synthetic preparation methods. In addition, nanoSiO_2_ is unintentionally present in silica powders typically used in the industry for food, cosmetic and health applications [7]. Given this wide range of applications, it is clear that intended or unintended exposure to amorphous nanoSiO_2_ is likely to occur, which depending on the route of administration and its physicochemical properties, may translocate into the bloodstream and become available to interact with tissues and organs. These possibilities are likely to occur when considering biomedical applications using SiO_2_, such as a recent diabetes clinical trial involving the consumption of 9 g of food grade SiO_2_ [1], where in some cases 33% of it can constitute nanoSiO_2_ [12].

The cardiac toxicity of amorphous nanoSiO_2_ in murine models has been previously studied. For example, intratracheal instillation of the nanoparticles was demonstrated to cross the alveolar-capillary barrier and impair vascular homeostasis, cause systemic inflammation [45], and a prothrombotic state [13]. Also endothelial and hemodynamic dysfunction in rats has been observed [19]. In adult rat cardiomyocytes, exposure to amorphous nanoSiO_2_ resulted in a functional loss of contraction caused by a dysregulation of intracellular Ca^2+^ handling, due in part to an increased oxidative stress production and reduced mitochondrial membrane potential (Δψ_m_), resulting in impaired ATP production [24]. In vitro studies have found that, when exposed to cardiomyoblasts, nanoSiO_2_ resulted in Cx43 phosphorylation, which led to an inhibition of the gap junction intercellular communication [14]. In HUVEC cells exposed to nanoSiO_2,_ a reduction in ATP content and reduced expression of genes related to mitochondrial biogenesis were found [26]. However, hitherto there is not enough evidence on how this could affect an intact heart, as well as an unclear picture on the role of nanoSiO_2_ and the specific toxicity mechanisms triggered on cardiac mitochondria.

Therefore, this study is focused on the deleterious effects of nanoSiO_2_ perfused into ex vivo rat hearts, and the assessment of its toxicity mechanisms in cardiac cells and isolated mitochondria of both rat and human cardiomyocytes. Results point towards a toxicity mechanism driven by the opening of the mitochondrial permeability transition pore (mPTP) through thiol oxidation of the adenine nucleotide translocase. These effects in the mitochondria were partially reversed using a potent antioxidant agent, and cellular cardioprotection was corroborated by mPTP blocking.

## Materials and methods

### Materials and reagents

Silica nanoparticles were AEROSIL 380 fumed silica. All reagents were obtained from Sigma-Aldrich (Sigma-Aldrich, St. Louis, MO) unless otherwise stated.

All the studies were performed in accordance with the animal care guidelines of the Guide for the Care and Use of Laboratory Animals, published by the National Institutes of Health (NIH Publication No. 85–23, Revised 1996). All procedures were approved by the Institutional Animal Use and Care Committee (protocol number 2017-Re-002).

#### Preparation of nanoSiO_2_

A general stock of nanoSiO2 was prepared in ultrapure H_2_O at a concentration of 10 mg/mL, sonicated during 30 min in a sonication bath. From this general stock, specific stocks were prepared for each experiment by diluting the general stock in the media, at the desired experimental dose(s), followed by vigorous vortex.

#### Particle size distribution (PSD)

Dynamic light scattering (DLS) was used to quantify the PSD of the nanoparticles (NPs) in aqueous solutions. Their PSD was obtained from the hydrodynamic diameter ensemble of the NPs, which was determined by fitting the measured intensity autocorrelation. Measurements were performed in a Malvern Zetasizer Nano ZS90 (Malvern Instruments, Malvern, UK).

#### Surface charge

Electrophoretic light scattering (ELS) was used to determine the zeta potential of the NPs dispersed in aqueous solutions through the Smoluchowski approximation. Measurements were performed in a Malvern Zetasizer Nano ZS90 (Malvern Instruments, Malvern, UK).

#### Specific surface area (SSA)

Nitrogen gas adsorption isotherm was measured using a sorptometer Quantachrome Autosorb-1 automated gas sorption analyzer (Quantachrome Instruments, Boynton Beach, FL). Samples (approximately 80 mg) were out-gassed overnight for 10 h at 250 °C before carrying out any measurements. A typical Brunauer–Emmett–Teller (BET) experiment was conducted to a relative pressure, P/P0 < 0.3 at 77 K, where P0 is the saturation pressure.

#### Ex vivo heart experiments

Male Wistar rats (250–300 g) were injected with heparin (1000 U/kg, i.p.) 20 min prior to anesthesia with pentobarbital (100 mg/kg, i.p.). Once bilateral corneal reflex was absent, the heart was excised through an abdominal approach. Afterwards, the ascending aorta was visualized and cut, placing the heart in a cardioplegic solution, which consisted in the sterile medium of a potassium chloride solution (in mM): NaCl 113, KCl 4.7, MgSO_4_ 1.2, Na_2_HPO_4_ 0.6, KH_2_PO_4_ 0.6, NaHCO_3_ 12, KHCO_3_ 10, Taurine 30, Hepes 10 and glucose 5; pH = 7.4 (adjusted with NaOH) and Osmolality = 302 mOsm; 1 mg/mL bovine serum albumin [31]. The time between cutting the diaphragm and placing the heart in the solution took less than 60 s in order to avoid ischemia. Hearts were mounted in accordance with the Langendorff model and perfused at a constant flow (12 mL/min) with a Krebs-Henseleit (K-H) buffer [11]. A latex balloon, connected to a pressure transducer filled with saline solution, was inserted into the left ventricle after establishing autonomous contraction. The pulmonary artery was cannulated and connected to a closed chamber using a Clark-type oxygen electrode (Yellow Springs Instruments, Yellow Springs, Ohio) to measure myocardial oxygen consumption (MVO_2_) in the coronary effluent. The rate of MVO_2_ was calculated as the difference between the concentration in the K-H buffer before (100%) and after perfusion. Data Trax software (WPI, Sarasota, Florida) was used for continuous recording of the heart rate (HR), left ventricular pressure (LVP), and maximum positive and negative derivative of left ventricular pressure (±dP/dt). The baseline was established during 10–15 min of K-H buffer perfusion, then either K-H buffer or K-H buffer + nanoSiO_2_ was continued to be perfused during 30 min, followed by 10 min of K-H buffer perfusion. Hearts were analyzed only if their basal left ventricular developed pressure was ≥ 80 mmHg. Experiments were done in the constant flow setting without external electrical pacing. The rate pressure product (RPP = HR × LVP) was evaluated afterwards [20].

#### Cardiomyocyte isolation

Wistar rats weighing 250–300 g were used to isolate cardiac cells. Animals were heparinized and anesthetized with pentobarbital sodium (1000 U/kg and 100 mg/kg i.p, respectively) before removal and hanging the heart. Following a gold standard technique [38, 60], hearts were mounted on a Langendorff apparatus and then perfused with Tyrode medium (TM) in mM: 128 NaCl, 0.4 NaH_2_PO_4_, 6 glucose, 5.4 KCl, 0.5 MgCl-6H_2_O, 5 creatinine, 5 taurine, and 25 HEPES, pH 7.4 at 37 °C for 5 min and digested by 0.1% collagenase type II (Worthington Biochemical, Lakewood, NJ) dissolved in TM for 15 min. Ventricles were dissected and cells mechanically disaggregated. Cardiomyocytes were washed in crescent concentrations of Ca^2+^ (0.25, 0.5, 1, and 1.5 mM) plus 0.1% albumin contained in the TM. Cells were used for experiments only if the isolation yielded at least 70% of rod shape cells.

#### Mitochondria isolation

Mitochondria were isolated from rat hearts as follows: heart tissue was minced and homogenized in cold mitochondrial isolation medium (in mM: 125 KCl, 1 EDTA, and 10 HEPES-HCl; pH 7.3). The mitochondrial fraction was obtained by differential centrifugation using the protease Nagarse, as previously described [10].

#### Presence of nanoSiO_2_ in heart and mitochondria

Electron microscopy was used to assess the presence of nanoSiO_2_ in heart tissue and in mitochondria of isolated cardiomyocytes. For nanoSiO_2_ in heart tissue, after ex vivo heart perfusion, a sample from the apex of the heart was taken for silicon (Si) quantification in a Scanning Electron Microscopy coupled with an Energy-Dispersive X-ray Spectroscopy detector (SEM-EDS) (Hitachi SEM1510). Analyzed areas were selected randomly. Samples were coated with a conductive layer previous to SEM-EDS measurements. For the assessment of nanoSiO_2_, in isolated cardiomyocyte mitochondria, cardiomyocytes were incubated during 24 h with 100 μg/mL of nanoSiO_2_. Then, they were detached from the laminin-coated cell culture plates, and prepared for Transmission Electron Microscopy (TEM) on grids using critical point drying with CO_2_. Samples were then stained with uranyl acetate and analyzed the ultrastructure of mitochondria. Presence of nanoSiO_2_ was observed in dark field as electron-dense spots and corroborated by TEM-EDS (JEOL 2010F).

#### Calcium retention capacity (CRC)

The CRC is a functional assessment of the sensitivity of the (mPTP) opening to mitochondrial Ca^2+^ overload and was evaluated by monitoring the absorbance of Calcium Green-5 N (CaG-5 N) as a Ca^2+^ indicator. Briefly, 300 μg of mitochondria were resuspended in 500 μL of respiration buffer (RB) containing in mM the following: 150 sucrose, 50 KCl, 2 KH_2_PO_4_, 20 Tris-HCl pH 7.3, 5 succinate, 2 μg/mL rotenone, 1 μM CsA, 0.3 μM CG-5 N salt-free (Thermo Fisher Scientific, Waltham, MA, USA). CG-5 N fluorescence (*λ*_ex_ 485 nm/*λ*_em_ 528 nm) was monitored at 25 °C at basal conditions, then 10 μM Ca^2+^ boluses were added. CRC was estimated as the sum of uptaken Ca^2+^, reflected by the decrease in fluorescence at each bolus. Experiments were done with constant agitation using a microplate fluorescence spectrophotometer Synergy HT (BioTek Instruments,Winooski, VT, USA).

#### Mitochondrial respiration and membrane potential (ΔΨ_m_)

Mitochondrial oxygen consumption rate (OCR) and membrane potential (ΔΨ_m_) were measured in parallel with an Oroboros Oxygraph-2 k. The experiments were carried out in respiration assay medium containing in mM: 125 KCl, 10 HEPES-HCl, and 3 KH_2_PO_4_ with pH 7.3. State 4 respiration was measured in the presence of 10 mM succinate-rotenone, and state 3 respiration was evaluated after addition of 100 μM ADP. Maximal respiration was determined with 0.08 μM of carbonyl cyanide m-chlorophenyl hydrazine (CCCP) [8]. The mitochondrial membrane potential was measured in parallel by fluorometry using 5 μM safranine [39]. Recordings were done simultaneously, mitochondrial respiration and *ΔΨ*_*m*_, during 15 min: 5 min exposed to nanoSiO_2_, followed by substrate-triggered mitochondrial activity during 10 min. The mitochondrial respiration, state 3 or 4, was analyzed as the maximal slope after addition of substrate. The *ΔΨ*_*m*_ was calculated as the maximal difference in arbitrary fluorescence between the stable state with succinate, and the addition of carbonyl cyanide-4-(trifluoromethoxy) phenylhydrazone (FCCP).

#### Oxidative stress

Mitochondrial oxidative stress was measured by aconitase enzyme activity and free thiol content. Posterior to nanoSiO_2_ exposure, and after a single Ca^2+^ bolus stimulus, similar to the procedure described in the CRC methods section, mitochondria were taken right after the treated group stopped mitochondrial Ca^2+^ transport, yet the control group was still transporting Ca^2+^ into the mitochondria.

Aconitase enzyme activity was measured by monitoring the rate of conversion of cis-aconitate, intermediate product from L-citrate at 25 °C at 240 nm using a spectrophotometer microplate reader Synergy HT (BioTek Instrument, Winooski, VT, USA) [65]. Briefly, 80 μg of isolated rat heart mitochondria were added to a total of 0.1 ml of respiration buffer (RB) with 0.01% Triton X-100 at pH 7.8. The reaction was initiated by the addition of in mM: 2 MnCl_2_ and 5 sodium citrate. An extinction coefficient for cis-aconitate of 3.6 mM^− 1^ was used to express the enzymatic activity as the formation of nmol cis-aconitate/min mg protein.

Free thiol content was measured by Ellman’s reagent, 5,5′-dithiobis (2-nitrobenzoic acid) (DTNB) as previously described [23]. In brief, 200 μg of isolated rat heart mitochondria were suspended in respiration buffer (RB), after 300 μM of DTNB was added and samples were incubated in dark for 10 min at 25 °C. Afterwards, samples were centrifuged at 10,000 rpm for 10 min. Absorbance was read using 100 μL of the supernatant at 412 nm. N-acetylcysteine was used as a standard.

#### Fluorescent labeling of thiols in mitochondria

Mitochondrial protein was concentrated to 10 mg/mL in Tris-HCl 0.2 M pH 7.2, EDTA 1 mM, SDS 1%, to expose all thiols to eosin maleimide labeling (100 mM, 10 min, at 4 °C in dark). Reaction was stopped by the addition of 50 mM DTT and further diluted in Laemmli buffer for SDS-PAGE separation (100 μg/lane). Fluorescence of labelled proteins were visualized using an UV transilluminator and the UVP image document system for the acquisition. Optical density from the 30 kDa band corresponding to the labelled ANT was normalized to the total protein load by Coomassie staining.

#### Cell culture, viability and oxidative stress assessments

Neonatal rat ventricular myoblast H9c2 cell line (CRL-1446) was purchased from ATCC (Manassas, VA, USA). Cells were grown in Dulbecco’s modified Eagle’s medium (DMEM, D7777) and supplemented with 10% fetal bovine serum (FBS) (Biowest, Riverside, MO, USA) and 1x penicillin-streptomycin (Gibco, Dún Laoghaire, Dublin, Ireland) in a humidified incubator at 37 °C with 5% CO_2_ and 95% air.

Cell viability was assessed by the Alamar blue viability test (Life Technologies, Carslbad, CA). In brief, H9c2 cells were seeded in 96-well plates at 1 × 10^4^ cells/well and 24 h later were treated with increasing doses of nanoSiO_2_. Viability and IC_50_ were assessed at 24, 48, 72, and 96 h in a microplate fluorescence spectrophotometer Synergy HT (BioTek Instruments, Winooski, VT, USA).

Cellular production of reactive oxygen species (ROS) by nanoSiO_2_ exposure, through hydrogen peroxide (H_2_O_2_) assessment were quantified in H9c2 cells, as recently reported [39]. Cells were stained with Amplex Red (Thermo Fisher Scientific). In brief, cells were detached with Trypsin (L0931, Biowest, Missouri, USA), then the cells were recovered by centrifugation and resuspended in a respiratory medium (in mM): 150 sucrose, 50 KCl, 2 KH_2_PO_4_, 20 Tris-HCl, pH 7.3 with 40 μM digitonin, 50 μM Amplex Red and 1.5 U/mL Horseradish peroxidase. Measurements were done using a microplate fluorescence spectrophotometer Synergy HT (BioTek Instruments,Winooski, VT, USA).

#### ATP measurement

ATP was measured using the Cell Titer Glow kit (Promega) in isolated ventricle myocytes (10^4^ cells) after 24 h exposure to nanoSiO_2_ at LD50, and in ventricle tissue after perfusion of nanoSiO_2_ in the isolated heart. Cells or N_2_ frozen-grinded tissue in p96 well format were lysed according to the manufacturer instruction to record the luminescence derived from the ATP-dependent luciferase activity, using a microplate luminometer. ATP was quantified using a standard curve and normalized to the total protein in the sample.

#### Measurement of oxygen consumption rates in human induced pluripotent stem cells cardiomyocytes

Oxygen consumption rates (OCR) were measured as previously described [52]. In brief, human induced pluripotent stem cells cardiomyocytes, which will be referred as human cardiac cells through the manuscript, were seeded at a density of 5 × 10^4^ cells/well in a Seahorse XF96 Cell Culture Microplate. Once attached, human cardiac cells were treated with 3 different concentrations of nanoSiO_2_ at 10, 100 and 500 μg/mL for 24 h and OCR was analyzed using a Seahorse XFe96 analyzer (Agilent Technologies, CA, USA). Prior to initiation of the assay, cardiomyocyte maintenance media was replaced with XF Media and during measurements of OCR, oligomycin (2.5 μM), FCCP (1 μM) and rotenone/antimycin A (2.5 μM) was sequentially injected into the system. The assay was repeated with two independent rounds of cardiomyocyte differentiation with each group consisting 11–13 wells. OCR readings were normalized to total protein content of each well.

#### Culture, differentiation and viability assessment of human induced pluripotent stem cells cardiomyocytes

Cardiomyocytes were derived from human induced pluripotent stem cells cardiomyocytes as previously described with modifications [28, 35]. Briefly, Human iPS-Foreskin-2 cell line, kindly provided by James A. Thomson (University of Wisconsin) [63], were maintained on vitronectin-coated plates in TeSR-E8 medium (Stem Cell Technologies, VA, Canada) according to the manufacturer’s protocol. For directed cardiac differentiation, iPSCs were dissociated into single cells and seeded onto Matrigel (Corning, MA, USA) coated plates at a density of 1 × 10^5^ cells/cm^2^ in TeSR-E8 medium supplemented with 10 μM Y^− 27,632^ (Tocris Bioscience, Bristol, UK). After 2 days when the cells were 100% confluent, which is referred to as day 0, medium was replaced with RPMI 1640 basal medium containing B-27 without insulin supplement (Thermo Fisher Scientific, VIC, Australia), growth factor reduced Matrigel (1:60 dilution) and 10 μM CHIR99021 (Cayman Chemical, MI, USA). After 24 h, medium was replaced with RPMI 1640 basal medium containing B-27 without insulin supplement for 24 h. At day 2, the medium was changed to RPMI 1640 basal medium containing B-27 without insulin supplement and 5 μM IWP2 (Tocris Bioscience) for 72 h. From day 5 onwards, cells were cultured in RPMI 1640 basal medium containing B-27 supplement (Thermo Fisher Scientific) and 200 μg/mL L-ascorbic acid 2-phosphate sesquimagnesium salt hydrate (Sigma-Aldrich). At day 12, cardiomyocytes were dissociated into single cells and seeded onto Matrigel coated 96-well plates at a density of 1.5 × 10^5^ cells/cm^2^ in DMEM/F-12 GlutaMAX medium supplemented with 20% fetal bovine serum (Sigma-Aldrich), 0.1 mM 2-mercaptoethanol, 0.1 mM nonessential amino acids, 50 U/mL penicillin/streptomycin and 10 μM Y^− 27,632^. From days 14–19, cardiomyocytes were enriched by culture in glucose-free DMEM medium containing 4 mM lactate (Sigma-Aldrich). Purified cardiomyocytes were maintained in RPMI 1640 basal medium containing B-27 supplement and 200 μg/mL L-ascorbic acid 2-phosphate sesquimagnesium salt hydrate.

Human cardiac cells were treated with increasing doses of nanoSiO_2_ in absence or presence of 0.5 μM CsA (Sigma-Aldrich) for 24 h. Cell viability was assessed by the Pierce LDH Cytotoxicity Assay kit (Pierce Biotechnology, IL, USA) according to the manufacturer’s protocol and by fluorescence microscopy as previously described [55]. For fluorescence microscopy, cells were stained with 1 μg/mL of propidium iodide and 3 μg/mL Hoechst 33258 at the end of 24 h treatment with nanoSiO_2_ for 15 min at 37 °C. The number of dead cells (as indicated by propidium iodide positive) was counted and expressed as a percentage over total number of cells (Hoechst 33258 positive). At least 300 cells were counted per group for each independent experiment.

### Statistical analysis

All measurements were performed at least in 3 independent experiments, reporting the average value and the standard error of the mean (SEM), unless otherwise stated. Statistical significance was compared between groups with an analysis of variance (ANOVA) followed by a Tukey or Kruskal Wallis post hoc analysis when appropriate.

## Results

### nanoSiO_2_ impairs relaxation on the ex vivo heart

The nanoparticles were characterized in terms of hydrodynamic diameter and ζ-potential in the Krebs-Henseleit buffer for the ex vivo experiments, following a specific inbubation protocol [24, 42]. The results are summarized in Table 1, showing a 3-fold agglomeration in the hydrodynamic diameter, while the ζ-potential remained mostly unaltered. This implies that the particles to which cardiac tissue was exposed in ex vivo experiments are an agglomeration of NPs. In addition, the SSA of the nanoSiO_2_ was assessed, resulting in 305.94 m^2^/g when analyzed by the BET theory or 470.50 m^2^/g by the Langmuir theory, differing from the reported SSA of 350–410 m^2^/g by the manufacturer.

**Table 1:** nanoSiO_2_ characterization in different aqueous media. Values are presented relative to those of nanoSiO_2_ in ultrapure H_2_O, 91 ± 22 nm for hydrodynamic diameter and − 27.1 ± 4.4 mV for ζ-potential, respectively as previously reported [24]

Media	Relative hydrodynamic diameter (nm)	Relative ζ-potential (mV)
Mitochondrial Respiration Buffer	0.73 ± 0.058	1.06 ± 0.122
Krebs-Henseleit buffer	2.99 ± 0.188	1.06 ± 0.122

**Fig. 1:**
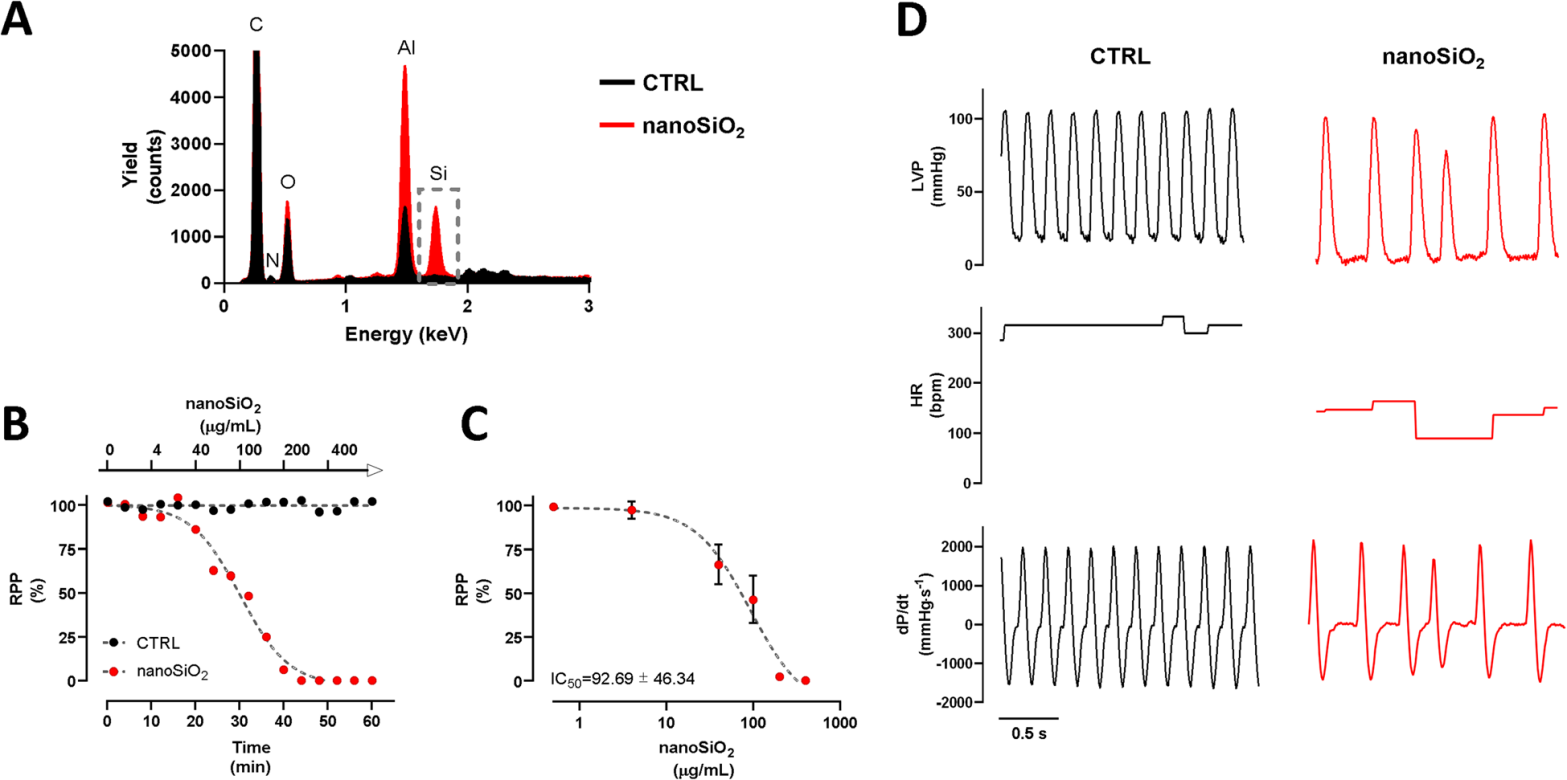
nanoSiO_2_ accumulates in heart tissue, diminishing contractility and affecting predominantly LVP and HR. **a** Silicon quantification in myocardial tissue by SEM-EDS after 100 μg/mL nanoSiO_2_ perfusion in ex-vivo heart. **b, c** Heart rate pressure product (RPP = HR × LVP) during 60 min after time- and dose- dependent nanoSiO_2_ administration. **d** The RRP dependence on nanoSiO_2_ administration reduced the frequency, and in some cases the amplitude of LVP and dP/dt, in addition a reduced HR. Values are percentage of control and represent mean ± SEM, *n* = 4

The effect of nanoSiO_2_ perfusion on cardiac function was studied in isolated rat hearts. Exposing the perfused hearts to 100 μg/mL resulted in accumulation on the apex, as evidenced in Fig. 1a. The RPP response was evaluated during 60 min using Krebs-Henseleit buffer with or without nanoSiO2, see Fig. 1b. The perfused group with nanoSiO_2_, with respect to the NT group, showed a sigmoidal dose-dependent activity with an IC_50_ of 92.7 ± 46.3 μg/mL, see Fig. 1c. Such reduced relaxation was reflected in the reduced HR of ~ 150 bpm in hearts treated with nanoSiO_2_ with respect to ~ 300 bpm of the NT group, see middle panels of Fig. 1d. Parameters like LVP and dP/dt were not affected in amplitude, see upper and lower panels of Fig. 1d. However, some LVP maxima were smaller for the nanoSiO_2_ treated group and their frequency were halved. Remarkably, relaxation rate (−dP/dt) was reduced 17%, suggesting an energetic impairment, as relaxation is the ATP demanding process in cardiac cycle. The results are summarized in Table 2. Under acute nanoSiO_2_ treatment, histopathology assessment of cardiac tissue after exposure did not reveal notable structural alterations, necrosis, signs of inflammation or rhabdomyolysis, suggesting that metabolic impairment proceeds to tissue disarrangement, see supplementary Fig. 1. Therefore, alterations in cardiac relaxation were found proportional to increased doses of nanoSiO_2_ perfusion, which may be associated with an increased NP accumulation in the cardiac tissue.

**Table 2:** Ex vivo perfused hearts with nanoSiO2, present reduced RPP, LVP and HR and –dP/dt

	Control	nanoSiO_**2**_	***p***-value
RPP (%)	99.4 ± 1.9	46.6 ± 13.6	0.009
LVP (mmHg)	105.3 ± 4	96.6 ± 4.4	0.194
HR (bpm)	313 ± 6	142 ± 14	< 0.001
+dP/dt (mmHg/s)	2018 ± 20	2049 ± 84	0.731
-dP/dt (mmHg/s)	− 1581 ± 12	− 1309 ± 62	0.005

### Isolated mitochondria exposed to nanoSiO_2_ present dysfunction due to the opening of the mPTP

Once the accumulation of nanoSiO_2_ on cardiac tissue and its effects on reduced cardiac relaxation were evidenced, direct exposure to mitochondria was studied in order to elucidate the toxicity mechanism. In cardiomyocytes, TEM micrographs present nanoSiO_2_ accumulation in mitochondria, see supplementary Figure 2. For the experiments with mitochondria, nanoSiO_2_ was characterized in terms of hydrodynamic diameter and ζ-potential in the respiration buffer, prepared similarly as the characterization for the ex vivo experiments. The results are presented in Table 1, showing a 27% reduction of hydrodynamic diameter, while the ζ-potential remained mostly unaltered.

**Fig. 2:**
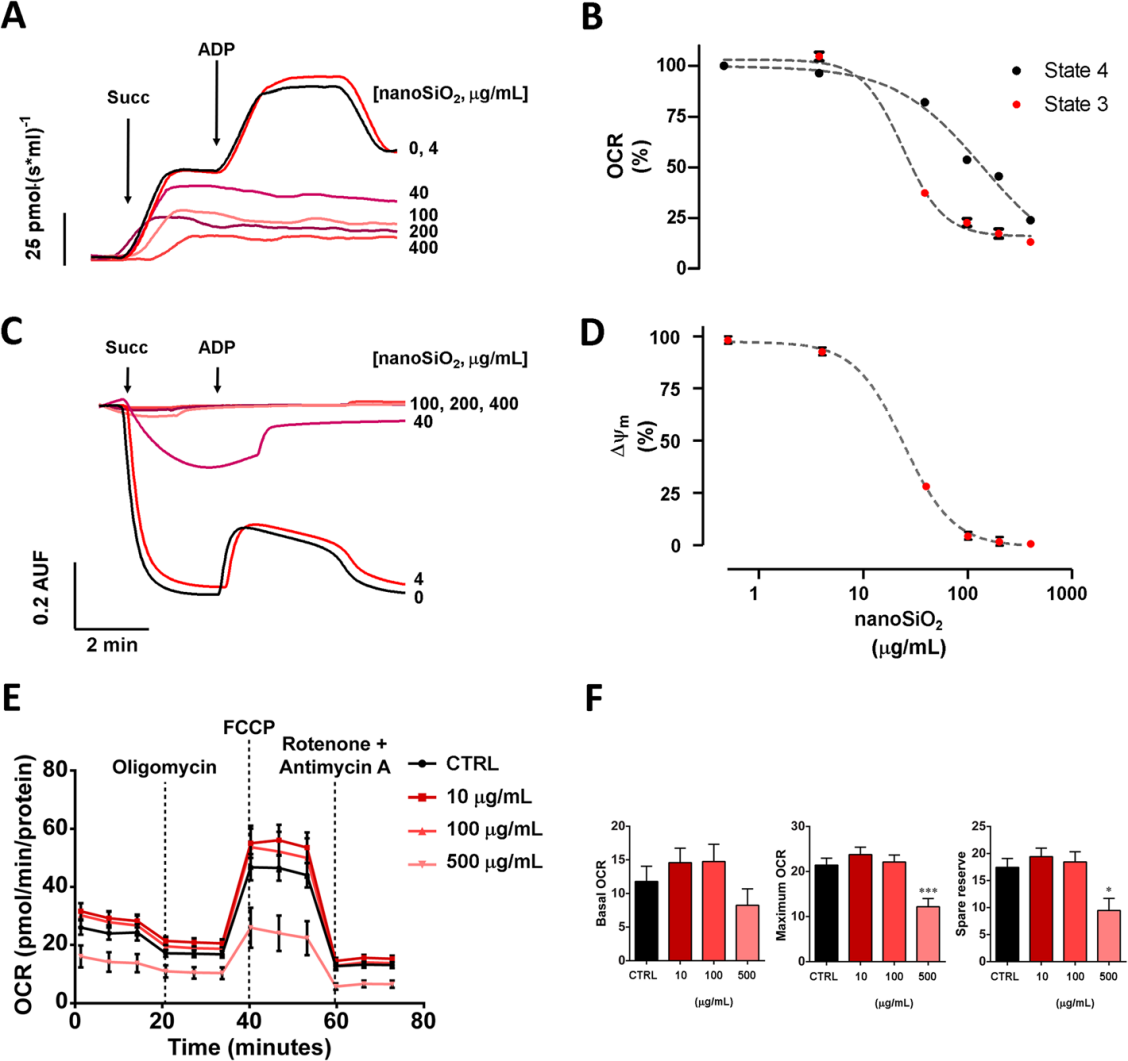
Exposure to nanoSiO_2_ to rat and human cardiac mitochondria results in mitochondrial dysfunction, observed by a reduced oxygen consumption rate and mitochondrial membrane potential. For rat cardiomyocyte mitochondria (**a-d**), at incremental nanoSiO_2_ concentrations: **a** Representative recordings of OCR. Addition of succinate and ADP are denoted by arrows. **b** Decrease of OCR evaluated in state 4 and state 3. **c** Representative recordings of ΔΨ_m_. Addition of succinate and ADP are denoted by arrows. **d** Decrease of ΔΨ_m_. For human cardiomyocyte mitochondria, at incremental nanoSiO_2_ concentrations: **e** Representative recordings of OCR. Addition of oligomycin, FCCP, rotenone and Antimycin A are denoted by dashed lines. **f** Basal and maximum OCR, and spare reserve. The exposure of mitochondria to nanoSiO_2_ was 5 min prior to measurements. Values represent mean ± SEM

**Fig. 3:**
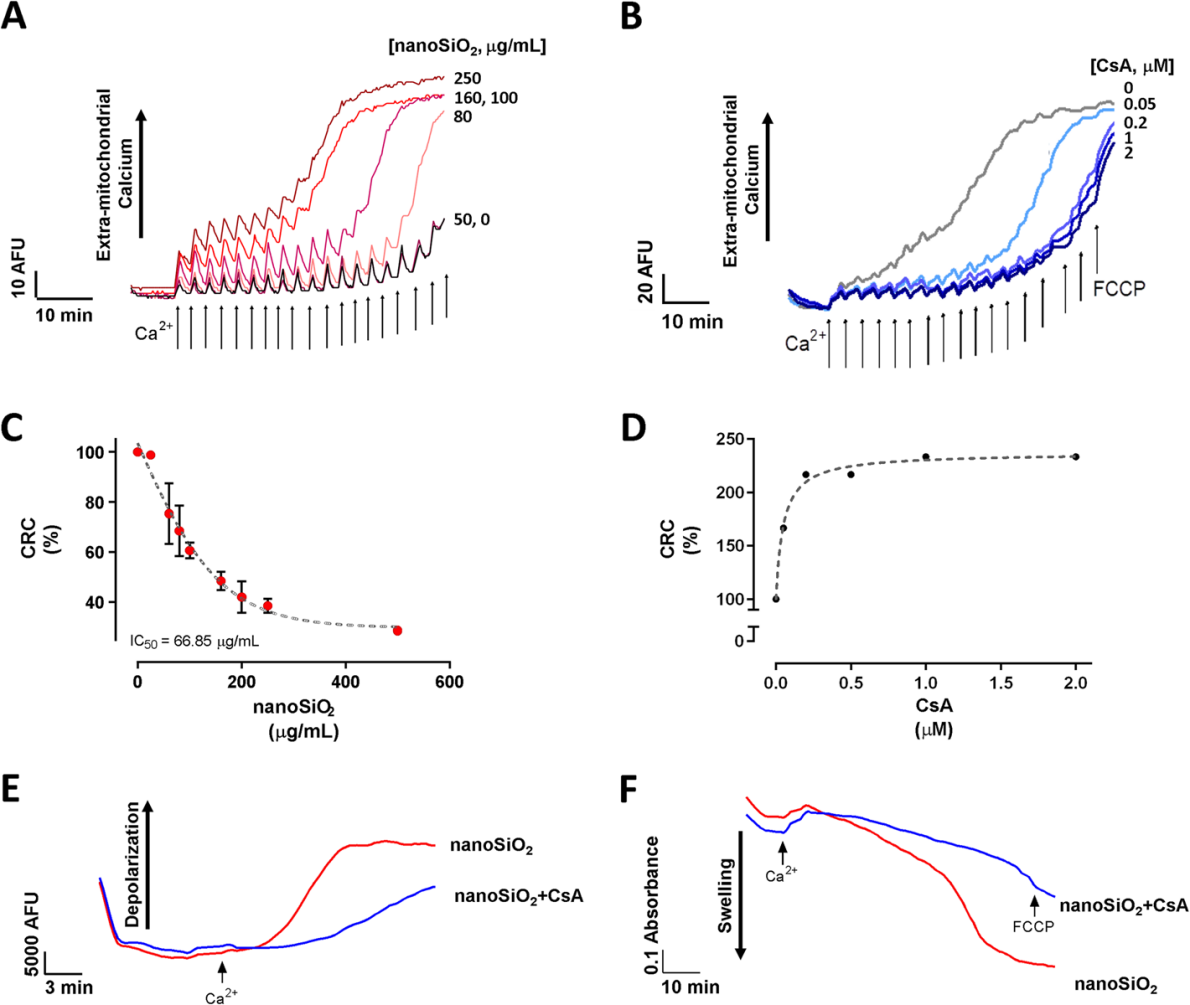
nanoSiO_2_ promotes mitochondrial membrane permeability associated to mPTP opening. **a**-**b** Representative recordings of mitochondrial CRC at increasing concentrations: (**a**) as a function of nanoSiO_2_, and (**b**) as a function of CsA. Arrows represent 10 μM Ca^2+^ bolus addition. **c**-**d** Semiquantitative analysis of CRC: (**c**) as a function of nanoSiO_2_, and (**d**) as a function of CsA. **e**-**f** Representative recordings of: (**e**) mitochondrial depolarization, and (**f**) swelling in presence and absence of CsA. The exposure of mitochondria to nanoSiO_2_ was 5 min prior to measurements. The concentration of nanoSiO2 in (**b**,**d**-**f**) was 100 μg/mL. CsA was applied at the same time of nanoSiO_2_ administration. Values are percentage of control and represent mean ± SEM

To this, isolated mitochondria from rat heart were treated with several doses of nanoSiO_2_, ranging from 1 to 400 μg/mL. The OCR was reduced directly proportionally to the applied dose, showing less sensitivity to the addition of substrates for ETC complex II, see Fig. 2a. State 4 presented an IC_50_ 140.9 ± 13.63 μg/mL versus of nanoSiO_2_, respectively; while state 3 showed a more sensitive decrease with an IC_50_ of 24.9 ± 7.5 μg/mL of nanoSiO_2_, respectively, see Fig. 2b. This reduction in OCR capacity with increased nanoSiO_2_ exposure was accompanied by a reduction in Δψ_m_, see Fig. 2c, presenting an IC_50_ of 24.4 ± 2.15, as shown in Fig. 2d. When mitochondria of human cardiomyocytes were treated with nanoSiO_2_, 500 μg/mL was found to have the most negative effect, 40% reduction, on oxygen consumption rate (OCR), see Fig. 2e. Although the reduction in basal OCR at 24-h post-treatment was not statistically significant, the maximum OCR (Untreated vs 500 μg/mL; 21.4 ± 5.50 vs 12.2 ± 6.00; *p* = 0.0009) and spare reserve (Untreated vs 500 μg/mL; 17.5 ± 5.80 vs 9.46 ± 7.43; *p* = 0.0104) was significantly reduced following treatment at 500 μg/mL in comparison to the untreated group, see Fig. 2f. Additionally, cardiac mitochondria exposed to increased doses of nanoSiO_2_ resulted with a lower mitochondrial calcium retention capacity (CRC), see Fig. 3a. The IC_50_ was estimated as 66.85 μg/mL, see Fig. 3c. The addition of a potent retardant of mPTP (CsA) partially recovered dose-dependent the CRC, about two-fold when 100 μg/mL of nanoSiO_2_ were administered, see Fig. 3b and d. The use of CsA delayed mitochondrial depolarization, as assessed by the ΔΨ_m_ in Fig. 3e, and reduced mitochondrial swelling as observed in Fig. 3f. These results indicate the mPTP may be associated in the deleterious effects caused by nanoSiO_2_.

### nanoSiO_2_ drive the mPTP through oxidative stress and thiol oxidation of the adenine nucleotide translocase

**Fig. 4:**
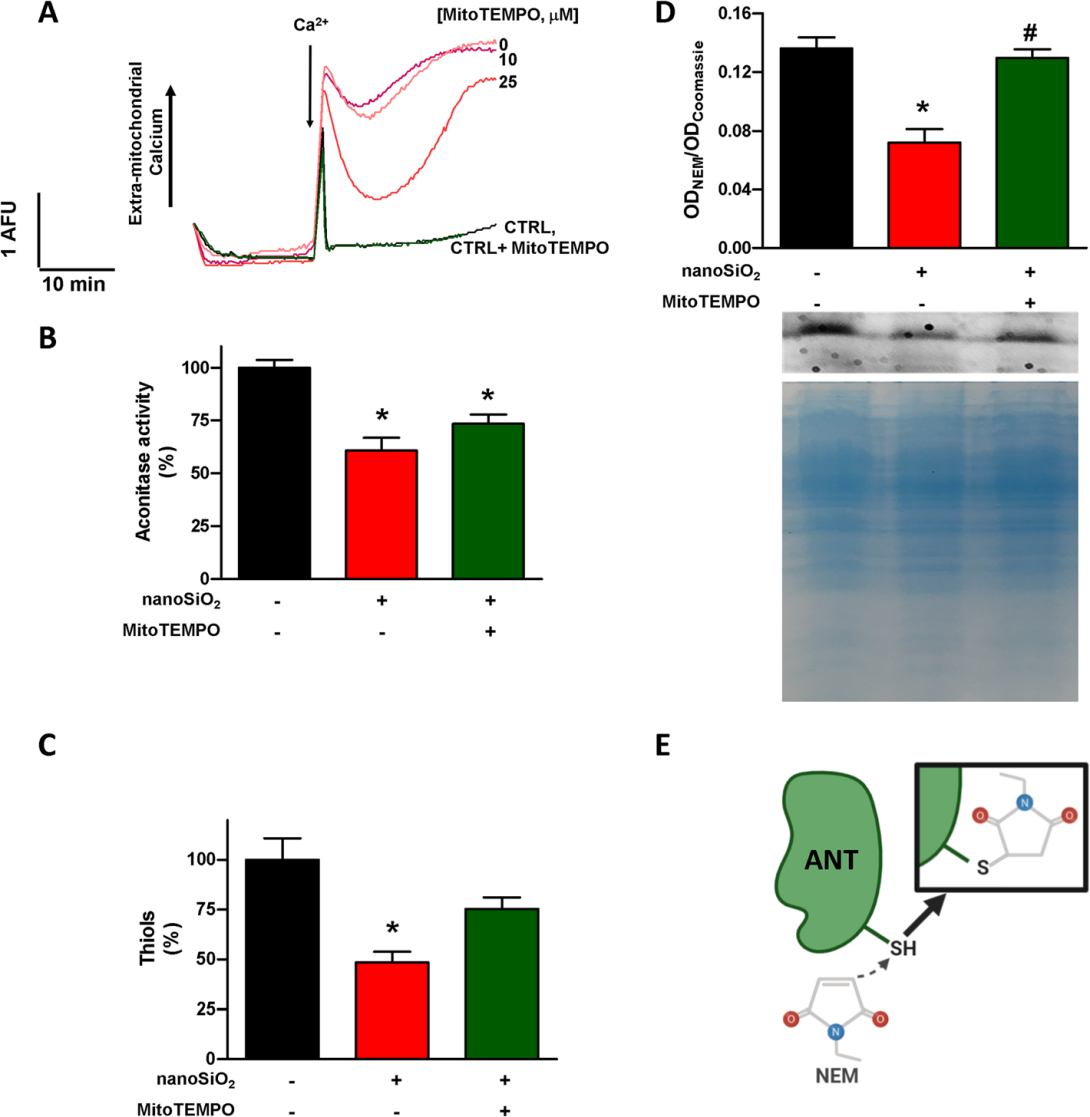
nanoSiO_2_ disturbs mitochondrial enzymatic activity and promote the oxidation of mitochondrial proteins. MitoTEMPO, a mitochondrial antioxidant, partially prevents nanoSiO_2_ oxidation effects. **a** Representative recordings of mitochondrial calcium transport in nanoSiO_2_ (30 μg/mL) incubated in mitochondria after the addition of 10 μM Ca^2+^. MitoTEMPO improved mitochondrial calcium transport. **b** Aconitase enzyme activity, **c** free thiol content, and **d** mitochondrial thiols in the ANT interlinked by n-ethylmaleimide (NEM) binding in isolated heart mitochondria after nanoSiO_2_ treatment (30 μg/mL) in presence or absence of MitoTEMPO (25 μM). The exposure of mitochondria to nanoSiO_2_ was 5 min prior to measurements. MitoTEMPO was applied 30 min prior to nanoSiO_2_ administration. Values are percentage of control and represent mean ± SEM. *p ≤ 0.05 vs control, #*p* ≤ 0.05 vs SNP. **e** Schematic interaction of NEM with SH groups in proteins

Mitochondria exposed to 30 μg/mL of nanoSiO_2_ induced the mPTP opening by Ca^2+^, as observed in Fig. 4a. In addition, aconitase activity was reduced by 41% after nanoSiO_2_ exposure, see Fig. 4b. Presence of thiol groups were similarly reduced 52%, see Fig. 4c. Finally, 53% less reduced thiol groups in the ADP/ATP translocase were found after NP exposure, see Fig. 4d. These results point towards oxidation damage as the toxicity mechanism of nanoSiO_2_ and confirm the involvement of the mPTP in this process. Given the damage may be mediated by the production of ROS, MitoTEMPO was used as a specific mitochondrial antioxidant agent, applied 30 min previous nanoSiO_2_ administration. The protective effect of MitoTEMPO on the mPTP was observed in a dose-dependent manner, see Fig. 4a. When nanoSiO_2_ triggered damage, mitochondria were treated with 25 μM of MitoTEMPO, finding that the aconitase activity, free thiols groups, and thiols in the ADP/ATP traslocase were partially recovered, see Fig. 4a-d, confirming the hypothesis that ROS is involved in the nanotoxicity of nanoSiO_2_. Such recoveries were correlated with a recovery in the mitochondrial selective permeability.

### Blocking the mPTP results in better cardiac cell fate

**Fig. 5:**
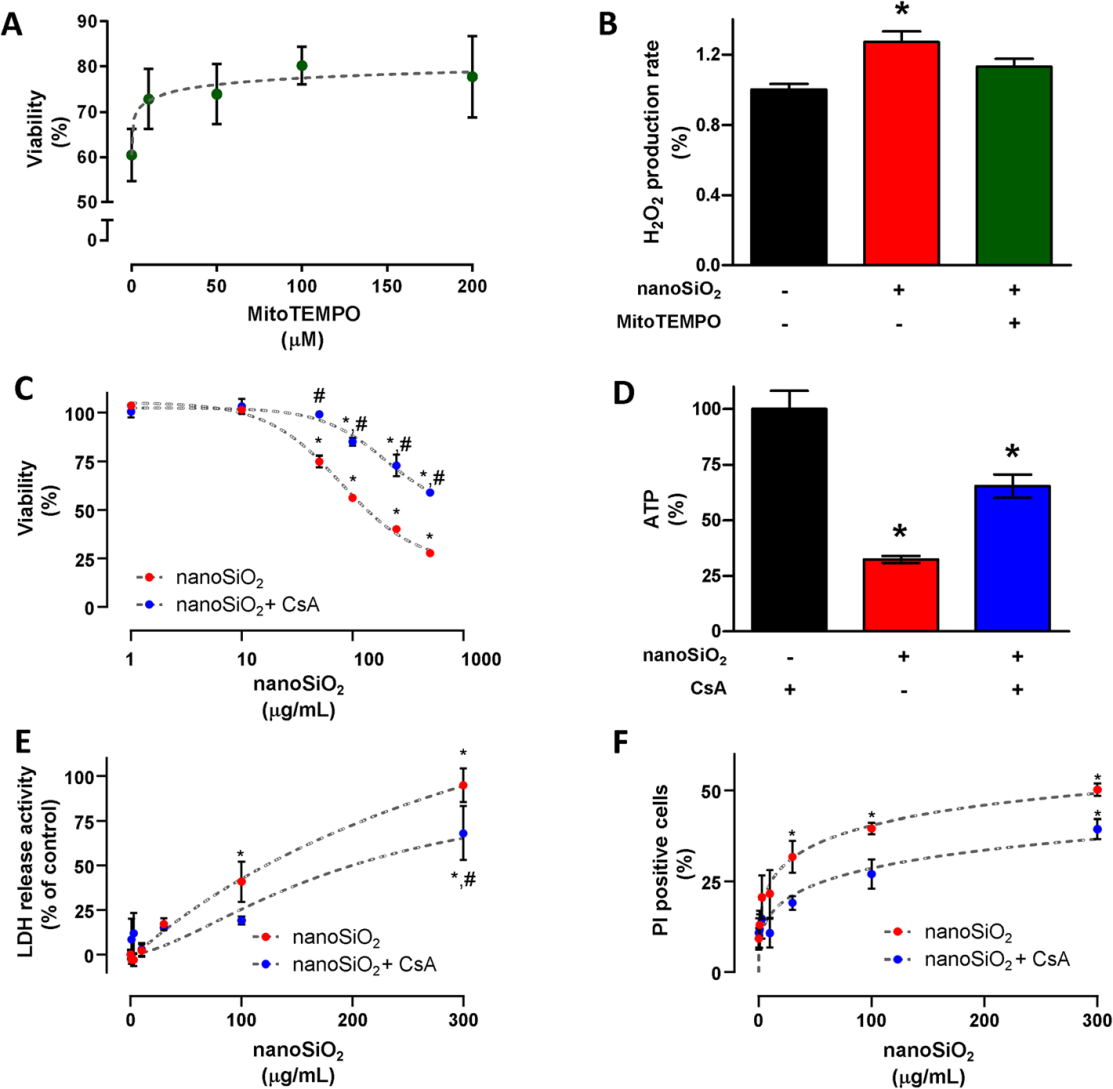
The toxicity mechanism of nanoSiO_2_ in cardiac cells is driven by reactive oxygen species and the opening of the mPTP. **a** MitoTEMPO dose-dependent cellular death prevention with 200 μg/mL of nanoSiO_2_ administration in H9c2 cells. **b** H_2_O_2_ production after nanoSiO_2_ administration (200 μg/mL) in presence or absence of MitoTEMPO (100 μM) in H9c2 cells. **c** Cellular viability in ventricular myocytes after nanoSiO_2_ administration in the absence or presence of CsA (0.5 μM). **d** ATP production in cardiomyocytes after nanoSiO_2_ administration (100 μg/mL) in absence or presence of CsA (0.5 μM). For human cardiomyocytes: (**e**) LDH release activity, (**f**) PI positive cells. MitoTEMPO or CsA were applied 30 min prior to nanoSiO_2_ administration. nanoSiO_2_ was incubated during 24 h. Values are percentage of control and represent mean ± SEM. *p ≤ 0.05 vs control, #*p* ≤ 0.05 vs CsA

In cardiomyoblasts, exposure to 24 h of nanoSiO_2_ resulted in improved viability when treated with MitoTEMPO, applied 30 min previous nanoSiO_2_ administration, see Fig. 5a. Treatment with ≥ 100 μg/mL of MitoTEMPO resulted in 33% higher viability. Similarly, H_2_O_2_ was exacerbated 27% with nanoSiO_2_, and was quenched to control levels with the addition of MitoTEMPO, see Fig. 5b. Exploring whether the blocking the mPTP with CsA would yield similar results to those of MitoTEMPO, cardiomyocytes were subjected to different doses of nanoSiO_2_, evaluating their viability 24 h later, see Fig. 5c. CsA was applied 30 min before administration of nanoSiO_2_. An IC_50_ of 79.7 ± 13.2 was found for nanoSiO_2_ exposure, which increased 2.5-fold when treated with CsA. ATP production, when exposed to the IC_50_ of nanoSiO_2_, was reduced down to 32.4%, and consequently was rescued significantly up to 67% when CsA was applied, see Fig. 5d. When human cardiomyocytes were treated with increasing doses of nanoSiO_2_, LDH release increased with higher NP doses, and similarly the onset of necrosis through PI staining commenced from 3 μg/mL and increased in a dose dependent manner, reaching 85 and 48%, respectively, see Fig. 5e and f, respectively. These results indicate that preventing the mechanisms of toxicity of nanoSiO_2_ in the mitochondria result in improved outcome for cardiac cells.

## Discussion

The toxicity of nanoSiO_2_ has been a concern due to their current wide industrial applications, such as an anti-caking agent in food products, and potential new areas such as biotechnological and biomedical applications. Amorphous SiO_2_ has been reported to elicit less toxicity in vitro or in vivo than its crystalline counterparts, and most nanotoxicity studies have been performed in amorphous nanoSiO_2_ [44]. Specifically, in cardiomyocyte research, nanoSiO_2_ has been reported only in relation to nanotoxicology rather than applications [40]. For NPs in general, particle size and particle number are the major drivers of a toxicological response. Regarding nanoSiO_2_, morphology and surface charge are the major toxicity response drivers [5]. A dominant factor in the toxicity of silica particles is the generation of ROS from the silica surface. In a recent study, Lehman et al. [36] reported that the amount of ROS species should be a function of the number of Si—OH. Therefore, toxicity of silica can be studied in terms of the surface chemistry, as defined by the assembly of siloxane (Si—O—Si) and silanol (Si—OH) groups that are bundled together to form a particle. A straightforward calculation for nanoparticles with a surface area of 380 m^2^/g (like those used in the present study) indicates that the concentration of hydroxyl groups is in the order of 10^4^ pmol OH/μg, based on a previous report of the of OH concentration in amorphous silica [49]. This result suggests that such a large amount of OH groups can be responsible for promoting several cellular reactions. For instance, the nucleophilic behavior of the silanol oxygen can also encourage the attack of electrophilic carbonyl groups, which are present in several proteins. Moreover, at physiological conditions (pH = 7.4) such terminal silanols can be deprotonated to form the silicate anion (Si—O^−^), which can further interact with cell membranes via electrostatic interactions [3]. Silica-derived ROS can lead to cellular oxidative stress that may be enhanced in the case it is nanosized owing to the greater surface area and therefore higher concentration of reactive groups. The increased production has been associated in vitro principally to an increased presence of ROS and through reduced GSH [24, 37], and producing cellular membrane damage through lipid peroxidation [2, 62]. In vivo exposure to nanoSiO_2_ is associated with inflammation through cytokine production and chemokine production [33]. It is worth noting that nanoSiO_2_ with a higher crystallinity, such as α-quartz, presents more toxicity than amorphous NPs [44]. This is likely due to the higher availability of silanol groups.

Exposure studies of nanoSiO_2_ in relation to its cardiovascular effects have been recently widely reported. For example, regarding in vivo studies, in old Sprague-Dawley rats, inhalation of 38 nm nanoSiO_2_ led to increased anoxic tissue, presence of troponin c in serum, and produced an incomplete atrioventricular heart block [6]. On the other hand, after nanoSiO_2_ intratracheal instillation, rats treated with 10 mg/kg of the nanoSiO_2_ (30, 60 and 90 nm) showed levels of high-sensitivity C-reactive protein and cytokines such as tumor necrosis factor-alpha (TNF-α), interleukin-1beta (IL-1β) and 6 (IL-6). Moreover, a significant decrease in nitric oxide production with elevated levels of intercellular adhesion molecule-l and vascular cell adhesion molecule-l suggested early steps of endothelial dysfunction [13]. Recently, it was observed that after nanoSiO_2_ intratracheal instillation, the descending aorta velocity was decreased 30%, concomitant with reduction in the cardiac output in nanoSiO_2_-treated group [19], indicating that nanoSiO_2_ in vivo alters cardiac hemodynamics. However, the precise mechanisms of this modification in cardiac output needs to be clarified. In this context, in zebrafish embryos pericardial edema and bradycardia were observed after a low dose intravenous injection of 106 nm nanoSiO_2_, due to downregulation of Ca^2+^ signaling genes [15]. VEGFR2-mediated autophagy in endothelial cells and pericytes was observed in ICR mice after a single intravenous dose > 100 mg/kg of 62 nm nanoSiO_2_ [16]. These mechanisms, in addition to apoptosis, were observed in Kupffer and HepG2 cells [64]. Similarly, in vitro, 24 h exposure of cardiomyoblasts up to 200 μg/mL of nanoSiO_2_ resulted in mitochondrial-mediated apoptosis along with a reduction in gap junction intercellular communication [14]. With respect to intracellular Ca^2+^ dynamics, adult rat cardiomyocytes exposed 24 h to 100 μg/mL nanoSiO_2_, of similar preparation and physicochemical properties as this study, resulted in alterations to Ca^2+^ handling and reduced cell shortening [24]. These changes were associated with increased intracellular H_2_O_2_ and reduced Δψ_m_ and ATP production. Similarly, ROS and mitochondrial dysfunction were observed in HUVEC cells exposed to 57 nm nanoSiO_2_ in 12.5–100 μg/mL, in addition to an observed reduced activity of the Na^+^/K^+^-, Ca^2+^-, and Ca^2+^/Mg^2+^- ATPases, mitochondrial fragmentation through the increase of FIS1/DRP1/Mfn2 and reduction of OPA1 proteins, and inhibited mitochondrial biogenesis via PGC-1a-NRF1-TFAM signaling [26]. To this body of knowledge regarding the toxicity cardiac impact of nanoSiO_2_, there is a lack of evidence regarding the functional impact of nanoSiO_2_ from a subcellular or organelle perspective, which could unveil the toxicity mechanism of nanoSiO_2_ that may link subcellular effects with cellular and tissue occurrences. Thus, the aim of this work is to present a mechanistic view on the effects of nanoSiO_2_ exposure directly on rat heart mitochondria, its effects on the function of ex vivo perfused and exposed rat hearts, and its possible mechanism of interaction with cardiac cells.

Reduced cardiac contraction, as observed in this study and others [19] due to exposure of nanoSiO_2_, can lead to reduced cardiac output. If this effect is sustained, then chronic cardiomyopathy is feasible, which may lead to heart failure. The amount of nanoSiO_2_ that reduced the relaxation of ex vivo hearts down to IC_50_ was estimated on average as 93 μg/mL, very close to the reported average of 99 μg/mL IC_50_ viability of adult rat cardiomyocytes [24]. Compared to other mitochondrial prooxidant molecules exposed under similar conditions to ex vivo hearts, the t_50_ present a similar value of 30 min [60]. Metabolic inhibition, either by depravation of substrates or reduction of the PO_2_ in the extracellular fluid, reduce the oxidative phosphorylation and the ATP availability. The sinoatrial node in the right atrium is the muscle cell mass responsible for the pacemaker activity. The resting membrane potential is rapidly recovered due, in part, to the opening of the K^+^ ATP (Kir6.X) channels, which may open after the energy-demanding relaxation where low ATP, or high intracellular ADP, activate the channels to repolarize [46]. Hypoxia and uncoupling of oxidative phosphorylation reduce the availability of ATP, increasing the current density of Kir6.2 that may lead to hyperpolarization and reduced excitability to decrease the fire rate in the pacemaker [4, 22]. This work shows that the direct administration of nanoSiO_2_ into the extracellular fluid of the isolated rat heart decreased in a dose dependent manner the RPP with a clear effect over the heart rate. Further examination demonstrated that nanoSiO_2_ accumulate within mitochondria to uncouple oxidative phosphorylation and reduce ATP availability. Hence, these events may have prompted the sustained opening of the Kir6.2 channels to reduce the heart rate, a survival mechanism described elsewhere during metabolic inhibition and ischemia [4, 25, 32]. Studies on the effects of cardiac contraction due to nanoparticle exposure are scarce [40]. In adult rat cardiomyocytes exposed during 24 h to the same nanoSiO_2_ as this study, a reduction in contractility, assessed by reduced cell shortening, was reported. The molecular bases of this contractile dysfunction are linked to longer SERCA Ca^2+^ recapture times, reduced Ca^2+^ sparks and specially a reduction in the Ca^2+^ content in the lumen of SERCA. This depletion of Ca^2+^ levels intra-reticulum is associated to a lower mitochondrial ATP production due to increased oxidative stress in the cells [24]. In zebrafish when exposed to nanoSiO_2_, a downregulation in genes of SERCA, calcium channel, and cardiac troponin C, as well as a decrease in the protein TNNT2 led the authors to speculate a potential reduction in cardiac contraction [15]. Recently, a study of nanoSiO2 exposure to Sprague-Dawley rats in a subacute administration via intratracheal instillation, found an increased dose-dependence effect in reduced ventricular function indicated by left ventricular ejection fraction and shortening [18]. These results are in agreement with the results here reported of dose-dependence ex vivo heart functional contractility. Reduction in contractility has been found with the exposure of other nanoparticles. For example, 24 h exposure to 100 μg/mL of 21 nm TiO_2_ NPs resulted in reduced contractility in adult rat ventricular cardiomyocytes [30]. Even short-term exposure to 25–35 nm TiO_2_ NPs in adult rat ventricular cardiomyocytes led to reduced contractility, yet showed increased spontaneous contractions [58]. Oral administration of colloidal Ag NPs in other animal experimental models also resulted in reduced myocardial contractility [51]. In spontaneously hypertensive rats exposed to subchronic intratracheal instillation of 25–35 nm TiO_2_ NPs, the isovolumic contraction time was found increased [56]. Therefore, it seems that a general consequence of inorganic NP exposure is the alteration of relaxation properties of the heart as we observed in this study. This is relevant to the proposed mechanism in this work, given that the process of relaxation and heart chamber filling is more energetically demanding and where alterations in mitochondrial function show with more clarity these phenotypes [54].

Mitochondria from heart tissue were exposed to nanoSiO_2_ for the first time in order to elucidate the underlying mechanisms through which the energetic state of rat cardiomyocytes deteriorated [24]. Impairment of OCR and reduction of ΔΨ_m_ was observed with a dose-dependent response at exposures higher than 1 μg/mL in rat cardiomyocytes. Similar effects were found on human cardiomyocyte mitochondria. The dissipation of the ΔΨ_m_, required for ATP synthesis, resulted in a reduction of 30% in ATP content with respect to untreated mitochondria. Such reduction in ATP is in line with previous reports, corroborating the alterations of the cardiomyocyte Ca^2+^ dynamics in active transport compartments [24]. These deleterious effects were at least driven directly by internalization of nanoSiO_2_ into the mitochondria. Uptake mechanisms of nanosized SiO_2_ have been reported through endocytosis [61] or passive diffusion [43], it could be speculated that nanoSiO_2_ uptake to mitochondria may be driven through passive diffusion. Their interaction with nanoSiO2 clearly showed a state of selective permeability loss of the internal mitochondrial membrane which explains the effect on the loss of ΔΨ_m_ and ATP synthesis. The effect of selective permeability was associated with the mPTP opening given that CsA addition resulted in a reduction on the ΔΨ_m_ loss and mitochondrial swelling. The partial reduction in mitochondrial selective permeability could be attributed to the internalization of nanoSiO_2_ which could be affecting it by interaction with the organelle with or without altering its structure [26].

The reasons for mPTP opening under nanoSiO_2_ exposure on mitochondria were explored, finding reduced aconitase activity and thiol content in mitochondria as the cause of oxidative stress. Aconitase is a well-known enzyme used to assess the damage by anion superoxide (O_2_^−^) [59]. On the other hand, total thiol groups decreased, suggesting there are processes related to cellular death, this is because thiol groups play a critical role in redox signaling by dragging between oxidized and reduced states in cardiac mitochondria [9]. In this regard, the oxidation of critical cysteines related with ANT thiol groups has been shown to be an important event of the Ca^2+^-induced mPTP opening. Here, using mitochondrial thiols interlinked by n-ethylmaleimide (NEM), it was observed a reduction in binding when nanoSiO_2_ triggered oxidative stress. This result indicates that the ANT-Cys^56^ has been affected by oxidation of thiols, because ANT-Cys^56^ is the site of interaction of NEM, modifying the translator in cardioprotective conformation to the mitochondrial matrix side that reduce the Ca^2+^-induced mPTP [27]. Remarkably, the mitochondrial antoxidant MitoTEMPO, under the same NP exposure conditions, delayed the opening of the mPTP and prevented the NT-Cys^56^ oxidation. These results make emphasis on the mechanism of mPTP opening, given that the ANT is one of the main components of the molecular structure of the mPTP [27].

**Fig. 6:**
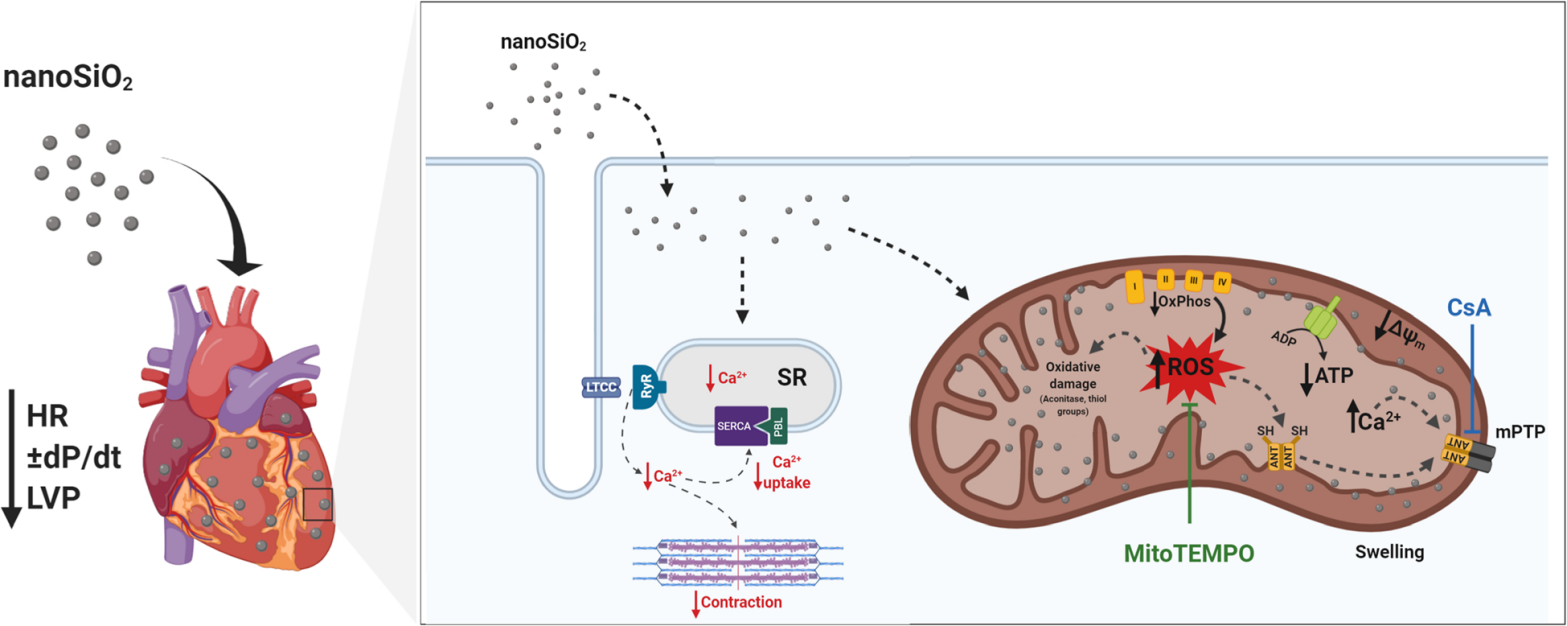
nanoSiO_2_ induces an increase in mitochondrial ROS production, leading to dysfunction in cardiac contractility. Hearts perfused with nanoSiO_2_ showed a compromised contractility, finding nanoSiO_2_ accumulation (heart representation, left side). Once nanoSiO_2_ internalizes into mitochondria, production of ROS is increased, compromising mitochondrial function. This leads to several oxidative damages, reducing the activity of the aconitase, and affecting the activity of key mitochondrial proteins such ANT through the oxidation of thiol groups. ANT oxidation promotes the mPTP formation, causing a decrease in mitochondrial membrane potential, which is the electrochemical force to synthetize ATP, compromising cellular viability. MitoTempo, a mitochondrial antioxidant agent, or CsA through delaying the formation of the mPTP, partially prevented these adverse effects of nanoSiO_2_ exposure

Given this oxidation mechanism and the mitochondrial protection using an antioxidant agent, it was sought to demonstrate whether such protection could be translated as in improved protection in a cellular model, to this adult rat cardiomyocytes or rat cardiomyoblasts were used and exposed to nanoSiO_2_ in the presence of MitoTEMPO. It was found that MitoTEMPO rescued viability 20%, and reduced H_2_O_2_. These results taken together points that cardiac tissue exposure to nanoSiO_2_ promote the oxidation of diverse mitochondrial components. In this sense it could be proposed the use of mitochondrial antioxidants directed towards cardiac tissue [39]. In adult rat and human cardiomyocytes, the use of CsA clearly improved cellular viability and rescued ATP production. Another strategy to reduce the effects of nanoSiO_2_ exposure may be the use of blockers of the opening of the mPTP, such as CsA which can be administered nanoencapsulated [29]. The proposed mechanisms of this work are summarized in Fig. 6.

Finally, it is of our concern the occupational exposure to NPs, especially those individuals with a cardiovascular disease. Recent work has shown that NP exposure exacerbates the cardiovascular damage, such as nanoSiO_2_ induced alterations associated to contractile dysfunction [15, 18] or pathophysiological conditions in which there is an increase of oxidative stress, such as myocardial ischemia or angina [39, 48]. In this context, the tissue of patients will have higher susceptibility, which could trigger not only a lower dose cytotoxicity, but will likely provoke a non-conventional toxicity mechanism in specific scenarios of the disease, for example a patient with heart failure or cardiac hypertrophy [21]. This is a relevant group given that it has been reported that their hearts can present enhanced extravasation and accumulation of NPs in the cardiomyocytes [57]. Considering this scenario, other cardiovascular conditions such as diabetic cardiomyopathy intensified their cardiotoxicity of metallic NPs which affected the perfusion pressure and LVP [53], as well as the exposure to TiO_2_ NPs in spontaneously hypertensive rats [56]. Based on this information, it is of utmost importance to develop in the near future studies that stratify the increased risk that patients with cardiomyopathies might present due to NP exposure.

It should be noted that the current study present limitations regarding the range of doses and exposure conditions. Firstly, doses of 100 μg/mL or higher of nanoSiO_2_ are unlikely to reach the bloodstream as an acute exposure, therefore the effects of this NP may not be as evident as here presented. Another aspect is the actual nanoentity that will interact with cardiac tissue regarding size and surface coating (the protein corona) based on the route of exposure [41]. Even at in vitro settings it has been demonstrated changes to the physicochemical properties of NPs [17, 24], or in or ex vivo settings as here reported. Nevertheless, exposure to high concentrations of particulate matter < 2.5 μm (PM_2.5_) increased hazard ratios for heart disease and other ailments [50], and recent reports indicate that silicon-based nanomaterials may accumulate 14-fold higher in in hearts with hypertrophy and pathological remodeling [57]. In this context the present study may provide insight regarding occupational exposure to industries that deal heavily with nanosized silica such as artificial stone [47], semiconductors [34], or the food industry [7]. For example, some of the results here reported show clear effects at low doses, such as the percentage of PI positive cells in human CMs (Fig. 5f). Therefore, experimental design of realistic exposure conditions may provide further information regarding risk assessment and the cellular mechanisms of action of nanoSiO_2_. In addition, studies blocking Ca^2+^ uptake to the mitochondria, such as blocking the mitochondrial calcium uniporter with Ru_360_, may provide a more detailed view of the role of the mPTP opening upon nanoSiO_2_ administration.

## Conclusion

Perfusion of nanoSiO_2_ NPs in ex vivo rat hearts resulted in a reduction of relaxation. In isolated mitochondria the interaction with nanoSiO_2_ generated a reduction in oxygen content and ΔΨ_m_ caused by the mPTP opening due to oxidation of thiol groups at the ANT. Studies in human cardiomyocytes corroborated the role of mPTP in the prevention of deleterious effects from nanoSiO_2_ exposure. Such alterations in mitochondria were partially recovered with the use of a mitochondrial antioxidant agent and its use on cardiac cells showed that cellular cardioprotection against the deleterious effects of nanoSiO_2_ might be achieved by preventing excessive oxidation of mitochondrial proteins and keeping the selective permeability of the internal mitochondrial membrane.

## Supplementary information

**Additional file 1: Supplementary Figure 1.** Perfusion for 30 min with nanoSiO_2_ did not cause structural alterations or inflammation in the heart apex. H&E staining of: A) untreated, B) 40 μg/mL nanoSiO_2_ perfusion, and C) 200 μg/mL nanoSiO_2_ perfusion. **Supplementary Figure 2.** nanoSiO_2_ accumulates in mitochondria from ventricle myocytes. Representative TEM micrograph of mitochondria showing swelling and its assessment by TEM-EDS from: (A) untreated rat CMs, (B) nanoSiO_2_ exposed rat CMs. (C) Quantification of Si content from EDS spectra.

## Data Availability

The datasets during and/or analysed during the current study available from the corresponding author on reasonable request.
