# Supplementary figures and images for: Amorphous SiO2 nanoparticles promote cardiac dysfunction via the opening of the mitochondrial permeability transition pore in rat heart and human cardiomyocytes
Source: Part Fibre Toxicol. 2020 May 7;17:15. doi: 10.1186/s12989-020-00346-2 (PMC7206702; doi:10.1186/s12989-020-00346-2)

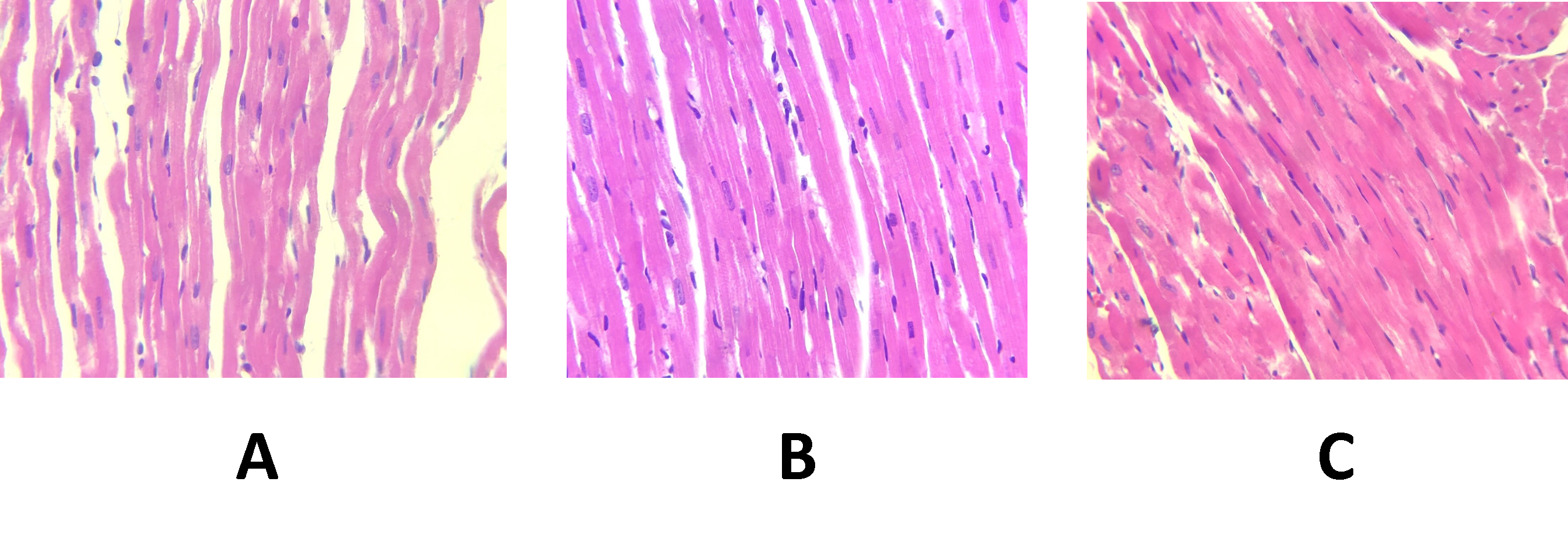

Supplement: Supplementary file 1 — Additional file 1: Supplementary Figure 1. Perfusion for 30 min with nanoSiO2 did not cause structural alterations or inflammation in the heart apex. H&E staining of: A) untreated, B) 40 μg/mL nanoSiO2 perfusion, and C) 200 μg/mL nanoSiO2 perfusion. Supplementary Figure 2. nanoSiO2 accumulates in mitochondria from ventricle myocytes. Representative TEM micrograph of mitochondria showing swelling and its assessment by TEM-EDS from: (A) untreated rat CMs, (B) nanoSiO2 exposed rat CMs. (C) Quantification of Si content from EDS spectra. [file 12989_2020_346_MOESM1_ESM.zip › Suppl Mat 1_ESM.tif]

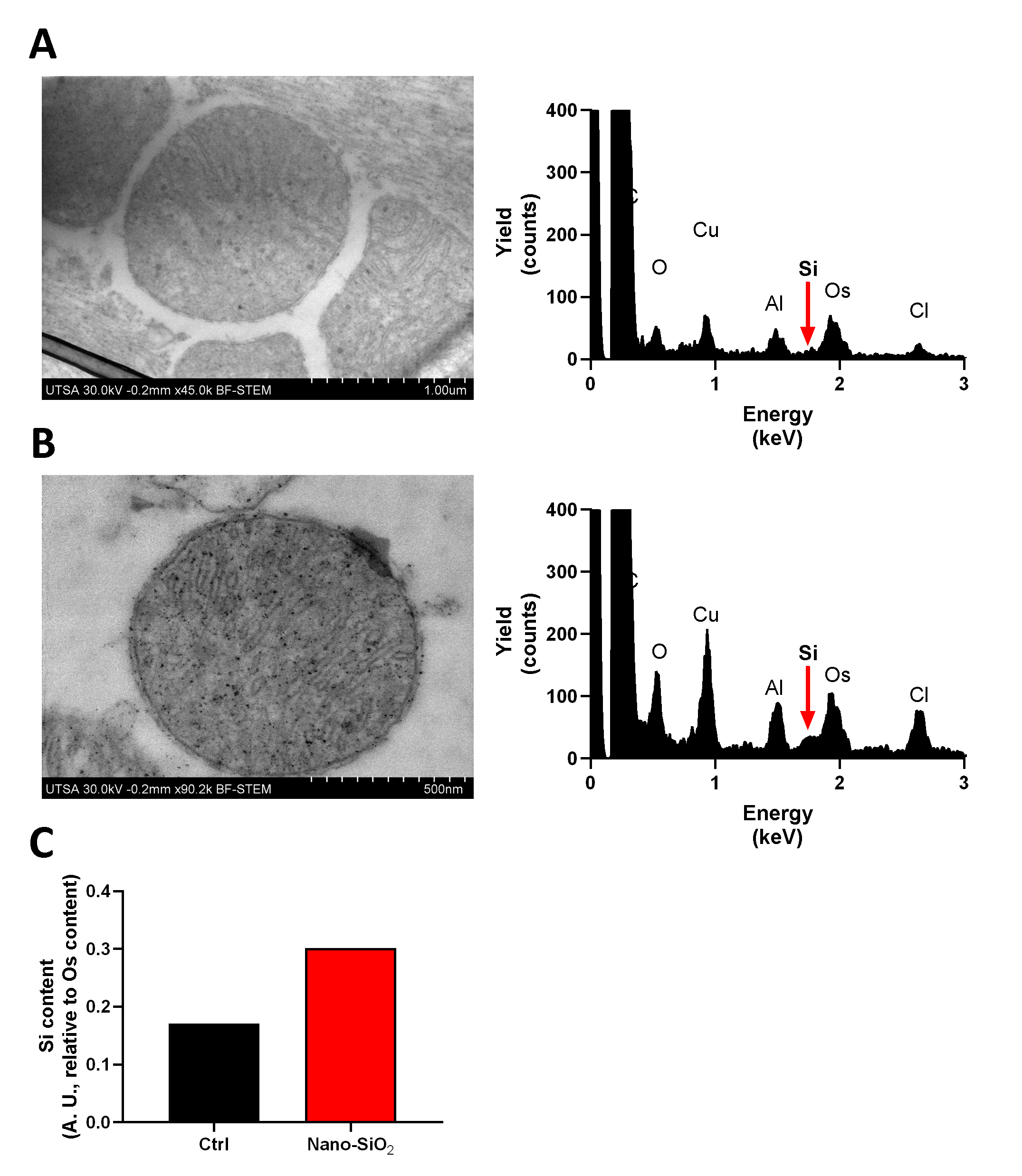

Supplement: Supplementary file 1 — Additional file 1: Supplementary Figure 1. Perfusion for 30 min with nanoSiO2 did not cause structural alterations or inflammation in the heart apex. H&E staining of: A) untreated, B) 40 μg/mL nanoSiO2 perfusion, and C) 200 μg/mL nanoSiO2 perfusion. Supplementary Figure 2. nanoSiO2 accumulates in mitochondria from ventricle myocytes. Representative TEM micrograph of mitochondria showing swelling and its assessment by TEM-EDS from: (A) untreated rat CMs, (B) nanoSiO2 exposed rat CMs. (C) Quantification of Si content from EDS spectra. [file 12989_2020_346_MOESM1_ESM.zip › Suppl Mat 2_ESM.tif]
